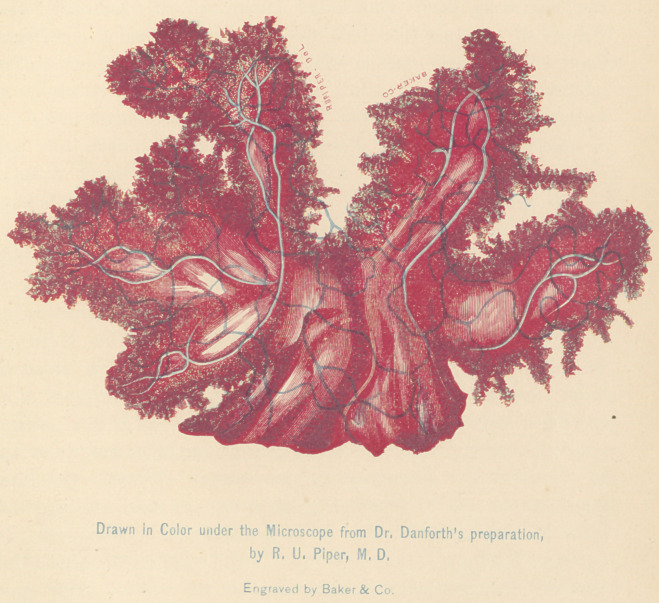# The Anatomy of Villous Cancer

**Published:** 1877-04

**Authors:** I. N. Danforth

**Affiliations:** Editor of the Pathological Transactions of the Chicago Medical Society


### THE ANATOMY OF VILLOUS CANCER.

(WITH PLATE)

BY THE EDITOR OF THE PATHOLOGICAL TRANSACTIONS OF THE CHICAGO MEDICAL SOCIETY.

In the *Chicago Medical Journal* for September, 1872, may be found an article by Dr. John E. Owens, concerning a case of cystic disease of the ovary under his care in St. Luke's Hospital. Ulcerative perforation of the intestine took place, which was promptly followed by general peritonitis and death. In pursuance of my duties as pathologist to the hospital, I made a *post-mortem* examination of the body, and my detailed report to Dr. Owens is incorporated in his article. The case proved to be one of those unfortunate ones which are necessarily fatal, and are therefore beyond hope of cure by any combination of surgical skill or experience; but it was a very fruitful and instructive one from the standpoint of pathology. Among other pathological products, I found one which I described as follows—quoting from the report aforesaid: "The uterus was slightly enlarged, the cervix filled with tough reddish mucus. From its posterior superior aspect, a little to the right of the median line, a cauliflower growth was found as large as a hen's egg; this proved to be a beautiful specimen of villous cancer." At that time I prepared and mounted some thin sections of this specimen, and they are yet in a good

Drawn in Color under the Microscope from Dr. Danforth's preparation,
by R. U. Piper, M. D.

Engraved by Baker & Co.

state of preservation, so that a very good idea of the peculiar architecture of villous cancer may be obtained by studying them. Believing that a brief description of the anatomy of this singular as well as rare form of carcinoma may be of interest to the members of the Society, and to the readers of its *Transactions*, I offer the following observations, and submit the accompanying colored plate. I ought, in the first place, however, to say a few words respecting the plate. It represents with great exactness the appearances of the specimen from which it was drawn. The section was stained with Beale's carmine, and mounted in Price's glycerine in a glass cell. Contrary to my usual experience with glycerine preparations, it has kept remarkably well, and may yet be studied with considerable satisfaction. The camera drawing was made in colors by Dr. R. U. Piper, and I have again to acknowledge my great indebtedness to his skill and kindness. The engraving was executed under Dr. Piper's personal supervision by Messrs. Baker & Co., of this city. It is a most successful reproduction of the appearances presented by the stained specimen from which the drawing was made. It represents the structure as seen under a magnifying power of 45 diameters. Of course it will be understood that it represents a carmine-imbibition, and not the natural color of the tumor. The so-called villous cancer of most writers upon pathology, is identical with the cylindrical epithelial cancer of Rindfleisch. It is very rarely seen except upon the surface of mucous membranes, and is then usually the product of long-continued irritative hyperplasia, most generally coupled with a constitutional tendency towards cancer—or a cancerous "diathesis." Its favorite locations are the mucous surfaces of the stomach and bladder—and of these, the bladder is most frequently invaded. "It occurs in its pure form," says Wagner; (General Pathology, page 492), "only on mucous membranes, (urinary bladder; uterus and vagina; so-called cauliflower growth of the vaginal portion of the uterus; stomach, etc.,) rarely in parenchymata." It is, in general, a modified form of papilloma, since it is developed from the same histological basis, namely, the epthelia of the papillar or glandular involutions of the mucous membranes or integuments. But, pathologically it differs radically from the simple papilloma, in that it results in the production of an atypical cell growth; hence it is truly a malignant growth.

But although villous cancer is pre-eminently a disease associated with the mucous membranes, it is well known that it *may* occur upon the integument and upon serous membranes. Upon this point Rokitansky says, (Path. Anat. Vol. 1, page 216): "So far as we know, it occurs solely upon membranes, for the most part, the pituitous, and most particularly upon that of the urinary bladder, as so-called villous muco-membranous tumor. It also, although far less frequently, affects the common integuments and serous membranes." In the case under consideration, it was produced upon the free surface of a serous membrane; that is the reflection of the peritoneum covering the posterior aspect of the fundus uteri. As the peritoneal membrane is destitute of both glandular involutions and villi, it follows that these structures are not necessary for the production of papillary tumors, whether malignant or benignant. Enquiry into the history of the patient failed to develop any constitutional tendency to cancer, or any local irritation or injury sufficient to account for the growth. Moreover, upon inspecting the body, I found no other cancerous deposits in any part or organ. The villous tumor connected with the uterus, then, was, so far as could be determined, a purely local manifestation, due to some entirely unknown cause. In the light of recent investigations in the domain of pathological cytology, we are warranted in presuming that the growth had its inception in some irritative disturbance of the nutrition of the part—that condition of things which Arnott has called "irritative hyperplasia."

The investigations of Klein (Stricker's Histology, page 572), seem to have demonstrated clearly enough that lymphatic canals or spaces exist in the serous membranes. This discovery renders it less difficult to account for the development of a villous growth upon the surface of the peritoneum, since it is now well known that the cells of the lymphatic vessels play an important part in the formation of cancerous structures. It seems now to be a pretty well settled doctrine of pathology, that cancerous new formations, whatever may be their variety or location, are the product of the atypical growth of epithelial cells. Of course this does not apply to the "small-celled infiltration;" but the small-celled infiltration cannot be regarded as a necessary element in cancer.

The specimen under consideration was, as I have described it, not far from the size of a hen's egg. It sprang from the posterior aspect of the fundus uteri by a large, short, stumpy pedicle; in fact, it would be just as accurate to say that it was sessile, and to regard the uterine or attached portion as the body of the tumor. The free surface presented the ordinary appearances of the more rapidly growing types of the papillomata; that is, innumerable papillæ or villi projected themselves in all directions from the body of the tumor, and reached out into the abdominal cavity. The surfaces of the mass of the tumor, and of the villous processes, were smooth and shining, and its color was identical with that of the neighboring peritoneum. The peritoneum, as far as the naked eye appearances indicated anything, seemed to be pushed forward, in advance of the sprouting mass, and therefore to cover each individual process which entered into the structure of the tumor. The growing villi underwent a process of subdivision, without any attempt at regularity or symmetry, until at length the ultimate villi were reached, and these were invariably clad with a luxuriant growth of cells, mostly club-shaped, or irregularly columnar; but these cells presented an endless variety, as regards form and size. That is, they were atypical epithelial cells; therefore they were typical cancer cells.

The plate represents a group of the larger villi, together with the pedicle by which they were attached to the body of the tumor. Their irregular or asymmetrical manner of subdivision is well shown. As the magnifying power was but 45 diameters, of course the minute structure of the ultimate villi is not shown—the design being rather to present a general idea of the architecture of the villous morbid growth. The vascular network was exceedingly rich. Only the larger vessels are shown in the figure; the darker network representing the subdividing arterial twigs, the light blue lines showing the returning venous trunks. When the margin of one of the outgrowths shown in the plate is examined with a half-inch objective, the rough, nibbled appearance seen in the figure is resolved into a luxuriant growth of primary villi. These minute offshoots present a very great variety, as regards form, size, and type. Some are long and slender, closely resembling the intestinal villi; some are shorter, and terminate abruptly in knobby extremities; some have very slender pedicles, and then suddenly bulge out into pouch-shaped extremities; some have very large pedicles, and as suddenly dwindle away to a terminal point. There are villi which have but a single terminal extremity, and those which have two or three; that is, those which divide dichotomously, or trichotomously. In short, it would seem as though every known—or, indeed, possible—variety of villus is utilized in the make-up of the villous cancers.

When a single ultimate villus is studied with a quarter-inch objective, it is seen to be clad with a dense, compact layer of irregular cells. A blood vessel enters it, forms a twisted and tortuous loop, and then emerges. The body of the villus seems to be made up of a closely woven mesh of connective tissue cells. On a future occasion, it is my purpose to present a plate, illustrating in detail, the structure of an ultimate villus.

Reported, March 19, 1877.

## Figures and Tables

**Figure f1:**